# Hypoxia-inducible factor-1 alpha modulates muscle growth and the molting process through its regulation of glycolysis in Neocaridina davidi

**DOI:** 10.1016/j.jbc.2025.110298

**Published:** 2025-05-27

**Authors:** Ran Li, Lezhen Hu, Runlin Zhou, Jialu Zhou, Jiale Yang, Moran Wang, Jinsheng Sun

**Affiliations:** 1 Tianjin Key Laboratory of Animal and Plant Resistance, College of Life Science, Tianjin Normal University, Tianjin, China; 2 College of Fishery, Tianjin Agricultural University, Tianjin, China

**Keywords:** HIF-1α, glycolysis, growth, oxygen, crustacean

## Abstract

Molting is a characteristic feature of crustaceans, closely associated with their growth. Following the molting process, crustaceans experience an explosive increase in muscle mass; however, the specific mechanisms underlying this rapid growth remain to be fully elucidated. This study aims to analyze the mechanisms of accelerated muscle proliferation from the perspective of sugar metabolism. The relationship between glycolysis and HIF-1**α** expression in shrimp muscle during different molting stages was investigated, revealing decreased glucose levels and elevated lactate concentrations in the D0-1 subphase. These findings suggest a Warburg-like effect occurring during pre-molting. Notably, HIF-1**α** expression was consistently higher in the D0-1, D2, and D3 subphases compared to other stages, indicating its pivotal role in muscle cell proliferation and molting regulation. Knockdown of HIF-1**α** significantly reduced the expression of glycolytic enzymes and inhibited both muscle growth and molting processes. Additionally, this study explored the effects of gill removal on HIF-1**α** expression; it was found that mechanical injury increased HIF levels, potentially mimicking hypoxic conditions. Specifically, tumor cell-free extracts were observed to enhance the molting rate, likely linked to upregulation of HIF expression. These results imply that such extracts may create a favorable environment for molting by influencing physiological mechanisms associated with HIF activity, thereby facilitating the molting process in shrimp. Overall, these findings suggest a complex interplay between glycolysis, HIF expression, and physiological processes during shrimp molting while highlighting the potential role of HIF as a regulator within these metabolic pathways.

Oxygen is essential to most living organisms, and its spatial and temporal availability in different environments has shaped the evolution of organisms and the organization of ecosystems (1). For example, aquatic environments have approximately 33 times less oxygen than terrestrial environments, primarily due to its comparatively low solubility in water. Furthermore, O_2_ is 10,000 times less diffusible in water than air (2). Many crustacean species that live in environments with fluctuating O_2_ deal with these variable conditions through morphological, physiological, and/or behavioral adaptations. Crustacean ventilatory and circulatory systems have biochemical and physiological mechanisms allowing them to respond to hypoxia. For example, the ventilatory rate and volume can be quickly regulated, which can impact the levels of O_2_ circulating in the animals. In the crayfish *Procambarus clarkii* and the rock lobster *Jasus edwardsii*, reduced levels of environmental O_2_ can reduce heartbeat rate (3) and ventilation rate (4). Furthermore, the presence of O_2_ sensors on the posterior gills is demonstrated by experiments in which animals with only one posterior gill (achieved through surgical removal of all other gills) exhibit a full cardiovascular response. This includes comparable cardiac output and heart frequency to that observed in intact animals (2).

In such a complex aquatic environment, completing molting and growth processes is a huge undertaking. Consequently, understanding how these organisms regulate their growth has been a topic of considerable scientific interest. The growth of crustaceans is discontinuous, with explosive muscle growth occurring following each molt. This accelerated growth necessitates the integration and coordination of various environmental factors sensed by molecular components within the body. Nevertheless, the molecular mechanisms underlying molting and growth remain largely elusive.

In the physiological investigation of rapid cell proliferation and growth, an intriguing area of focus is the process of glycolysis. As the primary process of sugar metabolism, it not only provides energy for cells but also contributes to the rapid growth of various organisms and cell lines. Notably, in the 1920s, Otto Warburg identified the phenomenon of aerobic glycolysis in cancer cells (5). Aerobic glycolysis is a metabolic transfer in which cells tend to convert glucose into lactate in the presence of oxygen and with normal mitochondrial function, accompanied by a metabolic phenotype of high glucose consumption rate and increased lactate production. Many researchers have found that normal cells also exhibit special phenotypes of increased glucose uptake and consumption as well as lactate accumulation. For example, it also occurs in many rapidly dividing cells such as highly active muscle contractions (6). Research reports that aerobic glycolysis takes place in the tail during early developmental stages as well as in mesenchymal cells exhibiting proliferation and differentiation capabilities in zebrafish (7, 8). The Warburg effect is also observed during the regeneration process of tadpole tails (9). The proliferation and division of progenitor cells in the embryonic retina of *Xenopus laevis* rely on aerobic glycolysis for RNA and protein biosynthesis (10). The enhancement of aerobic glycolysis is a typical phenomenon associated with lymphocyte division and transformation, while the transition to glycolysis in T lymphocytes coincides with their differentiation (11). Although there are few reports of aerobic glycolysis in arthropods, the rapid, almost 200-fold increase in body mass during the development of fruit fly larvae into adults is speculated to be driven by a Warburg-like mechanism (12).

In the process of embryonic myogenesis in *Drosophila* and zebrafish, the glycolysis genes phosphofructokinase (PFK) and phosphoglycerate kinase (PGK) are activated, which promotes an increase in muscle fiber size while facilitating the fusion of rapidly proliferating muscle precursor cells and myoblasts (13). Significantly, in *Aedes aegypti*, live bacteria or other living organisms (*e*.*g*. *Saccharomyces cerevisiae*) induce a hypoxia signal in the gut. This signal subsequently activates hypoxia-induced transcription factors and various processes essential for larval growth (14). Axenic larvae with no gut microbiota consume food similarly to conventional larvae; however, they fail to grow and die as first instars after several days. Larvae do not molt normally when exposed to environmental hypoxia, which suggests a requirement for localized microbe-induced hypoxia in the gut for normal growth and further supported that hypoxia leads to growth and ecdysone-induced molting (15). While aerobic glycolysis has been established as a crucial factor in cell proliferation across various species and cell types, it remains unreported whether such an effect is also present in the rapid growth of crustaceans. However, our previous published data have provided some insights (16); specifically, the mechanisms underlying muscle growth in crustaceans may exhibit similarities to those observed in fruit flies and mosquitoes.

There exists a pivotal transcription factor in the study of aerobic glycolysis known as HIF-1 (hypoxia-inducible factor 1). HIF-1 is ubiquitously found in animal cells, allowing these cells to sense and adapt to variations in oxygen levels while playing a crucial role in the regulation of oxygen homeostasis. There are two homologs of HIF-1 in mammals, HIF-2 and HIF-3, which together form the HIF family (17). Oxygen-sensitive alpha subunit and structural subunit aromatic hydrocarbon receptor nuclear transport factor 1 (HIF-1 β or ARNT1) heterodimerization to form HIF-1 (18). The activity of HIF-1 mainly depends on the regulation of HIF-1α, while the expression of HIF-1β is relatively stable. HIF-1 is a prominent and extensively studied transcription factor, with its structure and function being highly conserved across a wide range of species, from invertebrates and nematodes to vertebrates. HIF-1α can be activated by low oxygen levels, oxidative stress, growth factors, and various other stimuli. Research on mammalian HIF-1α has identified over 300 target genes, which primarily encompass a range of functions related to glucose metabolism (19, 20, 21, 22), vascular epidermal growth, and cell proliferation and apoptosis (23). HIF-1 can activate the transcription and translation of glycolysis-related genes in tumors, thereby driving the enhancement of aerobic glycolysis. Genes such as hexokinase (HK), PFK, PGK, and pyruvate kinase (PK) are its target genes (24), while glycolysis products pyruvate and lactate in turn promote the accumulation and stability of aerobic HIF-1α (25).

Long-term responses to O_2_ changes in levels in *Drosophila* require HIF prolyl hydroxylase as an O_2_ sensor (Katz *et al*. 2014; Pugh and Ratcliffe 2017). This enzyme is active under normoxia; non-naturally elevated levels of O_2_ are not required for its function. The consequence of elevated O_2_ levels is the inhibition of HIF-α activity, leading to a downregulation of downstream transcriptional events. Conversely, under hypoxic conditions, hydroxylation and subsequent degradation of HIF-α do not occur; as a result, HIF-α accumulates, dimerizes, and translocates into the nucleus where it functions as a transcription factor. In crustaceans, the hypoxia inducible factor-1 (HIF-1) signaling pathway is involved in hypoxia tolerance. In the muscles and gills of the crustacean *Penaeus vannamei*, 3-phosphoglyceraldehyde dehydrogenase (GAPDH) (26), hexokinase (27), and glycolysis are transcriptionally regulated by HIF-1 (28).

There have been numerous studies on HIF-1α as a transcription factor regulating aerobic glycolysis in mammals, but there are few reports on it in crustaceans. *Neocaridina davidi* exhibits remarkable adaptability to its environment and possesses a short molting cycle. Under optimal conditions, they molt frequently, making them an ideal experimental model for investigating molting and muscle growth in Decapoda shrimp and crabs. This study focuses on the key enzyme genes PFK, HK, and PGK that are involved in glycolysis within *N*. *davidi*. The effects of HIF-1α and the glycolytic pathway on both molting processes and growth phenotypes in shrimp were analyzed. This will help elucidate the regulatory mechanism of discontinuous muscle growth in crustaceans.

## Results

### The extent of glycolysis degree and HIF expression in the muscle of shrimp across various molting stages

**Figure 1:**
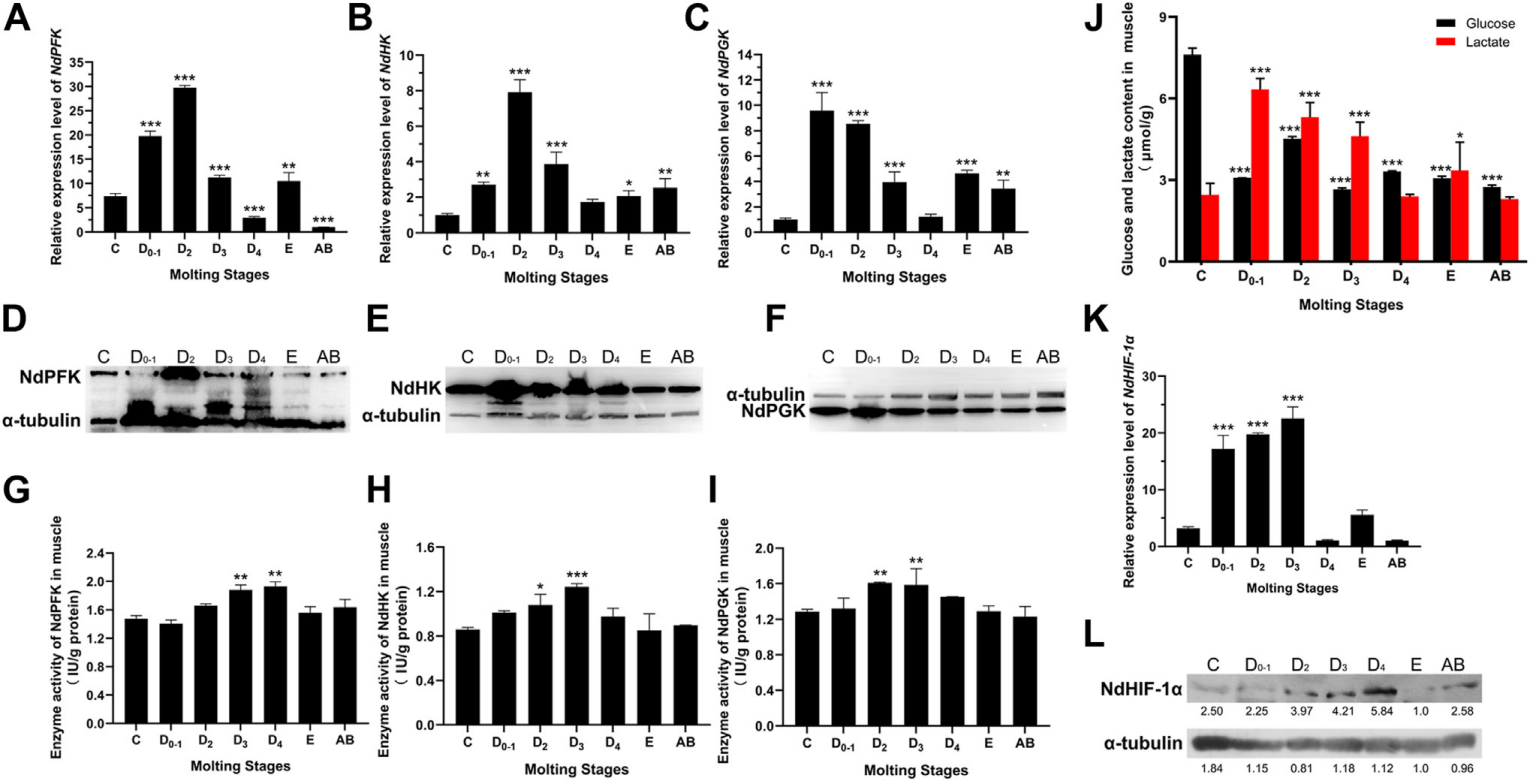
**Analysis of glycolysis among the molting process**. The relative expression levels of *NdPFK*, *NdHK*, and *NdPGK* genes in *Neocaridina davidi* muscle tissue across distinct molting stages are shown at both mRNA (*A*-*C*) and protein (*D*-*F*) levels. *C*, intermolt stage; D_0-4_: premolt stage; *E*: ecdysis; *AB*: postmolt. *G-I*, enzyme activity of NdPFK, NdHK, and NdPGK in *Neocaridina davidi* muscles among different molting stages. *J*, glucose (*Black* column) and lactate (*red* column) content in *Neocaridina davidi* muscle among different molting stages. Relative mRNA (*K*) and protein (*L*) levels of *HIF-1α* in muscles of *Neocaridina davidi* among different molting stages. The numbers below the blots represented the *gray* value as the fold-change relative to stage *E*. All stage groups were individually compared to stage *C* (designated as the reference control), with statistically significant differences (Dunnett's test: ∗*p* < 0.05, ∗∗*p* < 0.01, ∗∗∗*p* < 0.001) specifically marked by *asterisks* in the figures.

Fluorescence quantitative PCR was utilized to assess the transcriptional expression levels of NdPFK, NdHK, and NdPGK in muscle tissues at various molting stages, as illustrated in Figure 1, *A*–*C*. The durations of the sub-stages within the molting cycle exhibit variability. The C stage lasts 5 to 7 days, while the D_0-1_ stage spans 1 to 3 days. The D_2_ stage lasts about 1 to 3 days, and the D_3_ stage ranges from 16 to 24 h. The D_4_ stage is comparatively shorter, lasting around 8 to 12 h. In contrast, the E stage is notably brief, with a duration of merely 1 to 15 min; whereas the AB stage extends for approximately 12 to 24 h (29). The results revealed that the expression of these three enzymes exhibited consistent fluctuations throughout a single molting cycle. Specifically, NdPFK showed upregulation starting from the D_0-1_ subphase, peaking during the D_2_ subphase before experiencing downregulation in the D_3-4_ subphase. It then displayed slight upregulation during the E phase and reached its lowest expression level in the AB phase. Similarly, NdHK expression was found to be elevated during the early stages of molting but decreased significantly in the D_3_ subphase; no notable differences were observed between expressions in the D_4_ subphase and those in phases C and E. Conversely, NdPGK demonstrated its lowest expression level during phase C, followed by a sharp increase to its peak level at D_0-1_ subphase, subsequent downregulation thereafter leading to its minimum level observed during the D_4_ subphase. Western blot quantification revealed substantial concordance between transcriptional and translational peaks for the glycolytic enzymes: NdPFK protein abundance peaked during the D_2_ phase (Fig. 1*D*), aligning temporally with its mRNA maximum in D_2_, while NdHK exhibited overlapping expression surges in both mRNA (D_0-1_) and protein (D_0-1_-D_3_) profiles (Fig. 1*E*). NDPGK demonstrated strict synchronization between transcriptional (D_0-1_) and protein (D_0-1_) peaks. Overall, significant variations were noted in these three gene expression levels between the initial two substages of pre-molting compared to other stages. Further investigations were conducted to evaluate the enzyme activities of NdPFK, NdHK, and NdPGK within shrimp muscles across different molting stages (Fig. 1, *G*–*I*). The results revealed that activity patterns for these three enzymes initially increased before declining from phase C through phase AB. Notably, NdPFK exhibited peak activity during the D_4_ subphase while both NdHK and NdPGK reached their highest activities during their respective peaks at D_3_ and D_2_ subphases. Importantly, substantial changes occurred regarding enzyme activity across four substages of early molting; however, no significant differences were detected between inter-molting and molting phases.

To comprehensively evaluate the extent of glycolysis, we conducted measurements of glucose and lactate levels in muscle tissues across various molting periods. The results presented in Figure 1*J* indicate that overall glucose concentration is highest during Phase C, followed by a decline in the D_0-1_ subphase, with a slight increase observed in the D_2_ phase, leading to a general decrease during subsequent periods. In terms of lactate content, the initial three sub-stages of stage D exhibited significantly elevated levels compared to other molting stages. Notably, the D_0-1_ sub-stage demonstrated the highest lactate content, exceeding that of stages C and AB by factors of 2.6 and 2.8 times, respectively.

The presence of elevated glycolysis levels and the extensive expression of key enzymes, coupled with the close association between molting and vigorous growth, suggest a potential Warburg phenomenon similar to that observed in tumor cells during the early phases of molting. To investigate this hypothesis, we focused on a crucial molecule, HIF-1α, employing Quantitative PCR and Western blot techniques to assess NdHIF-1 expression in shrimp muscle tissue across various molting stages. Our findings, illustrated in Figure 1*K*, indicate that NdHIF-1α is expressed throughout all stages of molting, from interphase to late-stage molting. Notably, in the three sub-stages D_0-1_, D_2_, and D_3_ preceding molting, NdHIF-1 expression was significantly higher compared to the C, E, and AB stages. In contrast, expression levels during the D_4_ sub-stage were markedly lower than those observed in the preceding three sub-stages. During both inter-molting and post-molting periods, no significant differences were detected in NdHIF-1 expression. Immunoblot analysis revealed that HIF protein levels were significantly elevated during the D stage relative to other stages. Specifically, HIF expression peaked at the D_4_ stage within this phase (Fig. 1*L*).

### The impact of NdHIF-1*α* knockdown on glycolytic target enzymes

**Figure 2:**
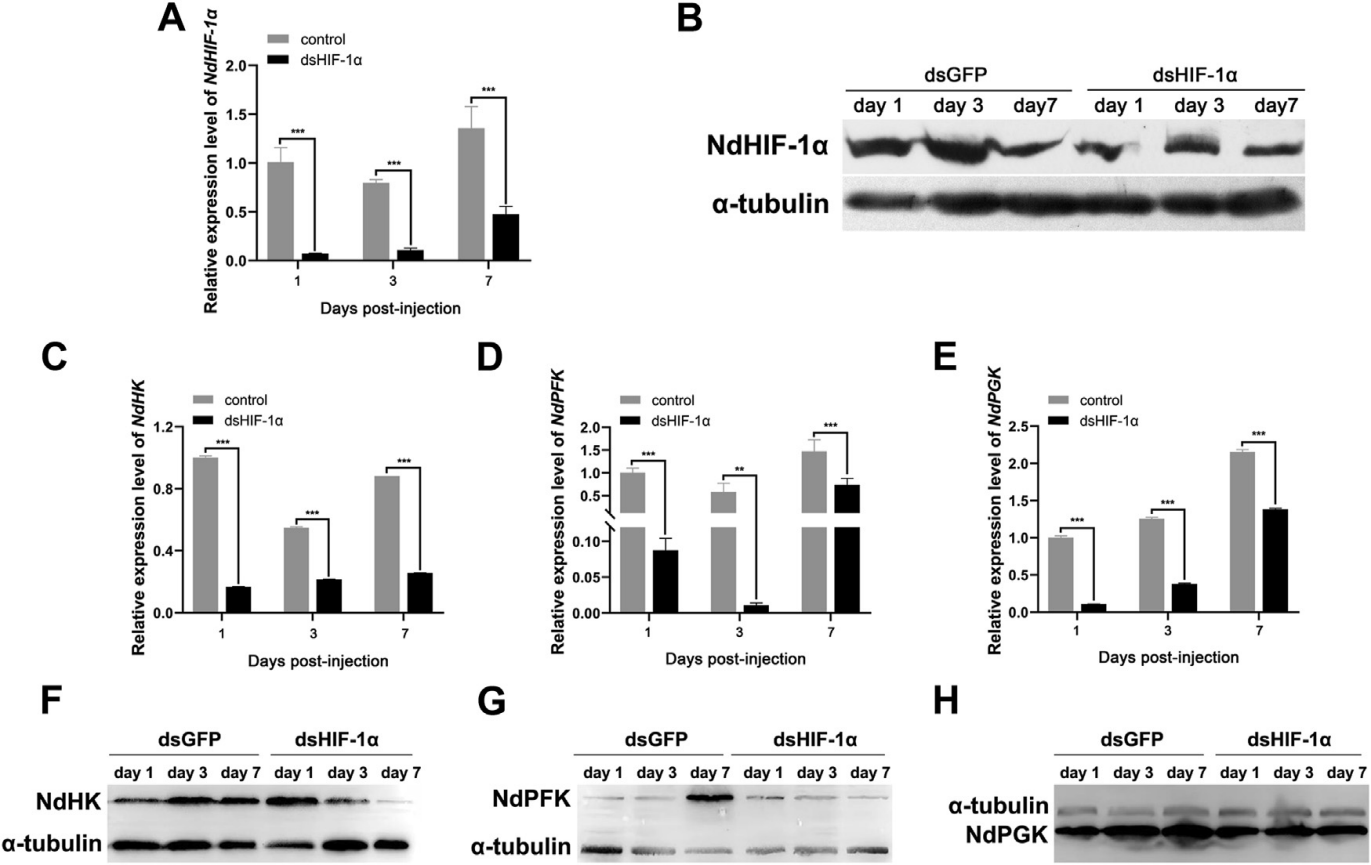
**RNAi of *NdHIF-1α***. The relative mRNA (*A*) and protein (*B*) expression change of *NdHIF-1α* over time in muscles after injection of double-stranded RNA. Significant differences (∗*p* < 0.05, ∗∗*p* < 0.01, ∗∗∗*p* < 0.001) between groups were indicated. Relative mRNA (*C*-*E*) and protein (*F*-*H*) expression of *NdPFK*, *NdHK*, and *NdPGK* over time in shrimp muscles was shown following dsRNA injection.

The expression levels of mRNA and protein for NdHIF-1α were evaluated in both the control and experimental groups of shrimp muscles on the first, third, and seventh days post-injection. The results, illustrated in Figure 2, *A* and *B*, indicate a significant reduction in HIF-1α expression within the experimental group compared to the control group. Specifically, the knockdown effect led to decreases of 92.95%, 86.4%, and 65% after 1, 3, and 7 days, respectively, with the most pronounced impact observed within the first 24 h following intervention.

Quantitative PCR (Fig. 2, *C*–*E*) was utilized to analyze alterations in transcriptional levels of glycolytic enzyme genes NdPFK, NdHK, and NdPGK in shrimp muscle from both interference and control groups at three time points (1 day, 3 days, and 7 days). The findings demonstrated a significant downregulation of these three genes when compared to controls across all time points assessed. Notably, NdPFK exhibited the most substantial downregulation after a 3-day interference period (Fig. 2*B*), while both NdHK and NdPGK reached their lowest expression levels after just 1 day of interference. To further validate these findings, we performed protein-level detection (Fig. 2, *F*–*H*). The results indicated that following the injection of dsHIF1-α, the levels of NdHK, NdPFK, and NdPGK proteins decreased over time in comparison to the control group. This observation further supports the notion that HIF knockdown inhibits the expression of these three proteins.

### NdHIF-1*α* knockdown caused inhibition of muscle growth and molting

**Figure 3:**
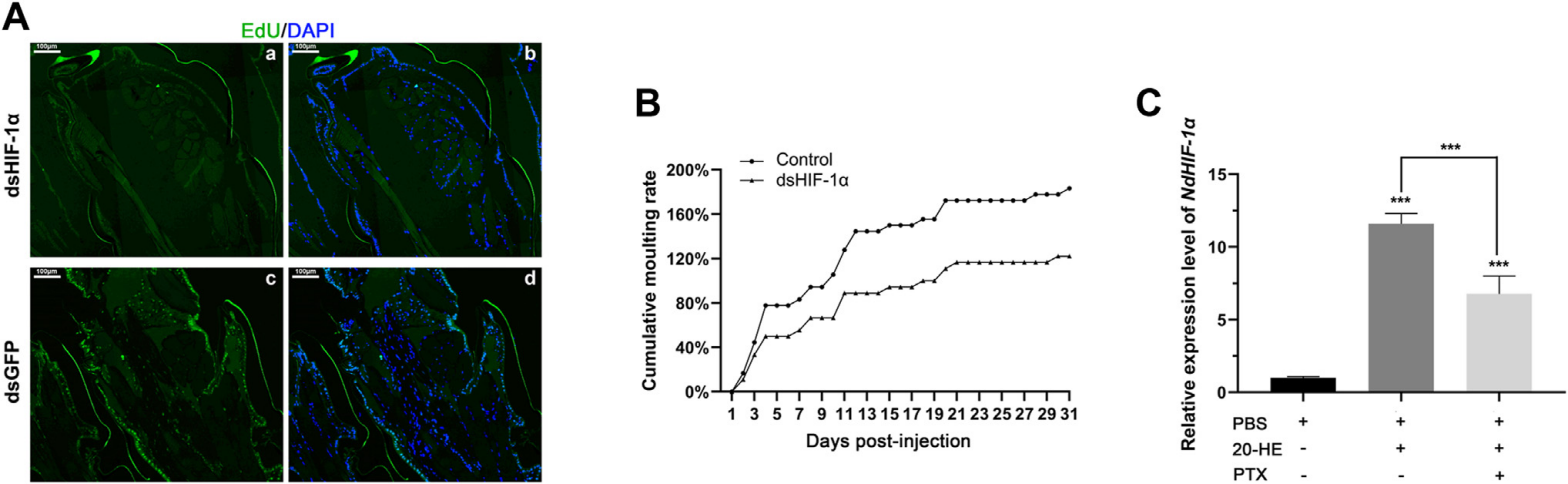
**The impact of HIF-1α knockdown on muscle development and molting in *N*. *davidi***. *A*, following interference with GFP and HIF-1α, EdU and DAPI staining were conducted on the appendage muscles to assess cellular proliferation and nuclear integrity. *B*, statistics on the number of molting cycles completed in RNAi interference and control groups. *C*, the relative expression of *NdHIF-1α* in muscle at 8 h after injection of different reagents. Statistically significant differences between all pairwise group comparisons are denoted by asterisks (∗*p* < 0.05, ∗∗*p* < 0.01, ∗∗∗*p* < 0.001). 20-HE: 20-Hydroxyecdysone; PTX: GPCR inhibitor pertussis toxin.

The preceding findings provided initial evidence for the presence of physiological mechanisms similar to the Warburg effect during the molting process of crustaceans, particularly in its initial phase. Therefore, it was hypothesized that the suppression of HIF activity may lead to a reduction in cellular proliferation and growth. Examination of appendage muscle sections from two cohorts of shrimp, which were concurrently administered EdU and dsGFP/dsHIF (as illustrated in Figure 3*A*), revealed a specific percentage of EdU-positive cells in the control group, indicating active muscle cell division and proliferation. In contrast, the experimental group of shrimps subjected to HIF-1α interference exhibited almost no EdU-stained positive cells within their appendage muscles. These findings suggested that HIF-1α played a crucial role in regulating shrimp muscle cell proliferation. Following this interference, shrimp were cultured and allowed to molt over 1 month (Fig. 3*B*). In the control group, all shrimp completed one round of molting within 10 days; however, it took 20 days for the experimental group to complete a single molting cycle. After 1 month, the number of molting cycles completed was recorded at 180% for the control group while remaining below 120% for the experimental group. These results indicated that HIF-1α not only influenced muscle proliferation during early stages of molting but also affected overall molting processes.

Molting progression in crustaceans is coordinated through neuroendocrine signaling, involving both neuropeptides (*e*.*g*., molt-inhibiting hormone (MIH) and steroid hormones (*e*.*g*., ecdysone). Given that our results highlighted HIF-1α as an integral component of physiological processes involved in molting, we sought to determine whether it is regulated by these hormones. Shrimp were injected with ecdysone alongside G Protein-Coupled Receptor (GPCR) inhibitor pertussis toxin (PTX), followed by assessment of HIF-1α expression levels. The results demonstrated (Fig. 3*C*) that, compared to the control group injected solely with PBS, there was a significant increase in the expression of HIF-1α in the ecdysone-only injection group. When ecdysone and PTX were administered simultaneously, the expression level of HIF-1α was markedly higher than that observed in the control group; however, it remained significantly lower than that seen in the ecdysone-only injection group. These findings suggest that the regulation of muscle cell proliferation and the molting process by HIF-1α is intricately coordinated and modulated by both ecdysone and molting-related neuropeptide hormones.

### The statistical analysis of heart rate, gill ventilation, and dissolved muscle oxygen concentration at various molting stages of shrimp

**Figure 4:**
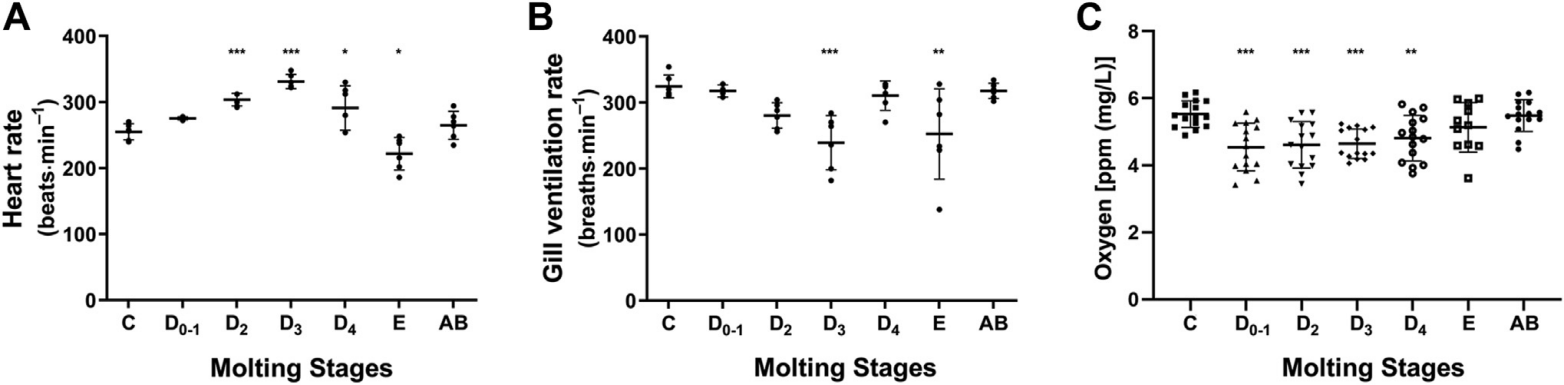
**Investigation of physiological characteristics in shrimp throughout various molting stages.** Heart (*A*) and gill ventilation (*B*) rate of *N*. *davidi* among different molting stages. *C*, temporal variations in oxygen content within *Neocaridina davidi* muscle tissue across distinct molting stages. All stage groups were individually compared to stage *C* (designated as the reference control), with statistically significant differences (Dunnett's test: ∗*p* < 0.05, ∗∗*p* < 0.01, ∗∗∗*p* < 0.001) specifically marked by *asterisks* in the figures.

The findings presented earlier prompt an inquiry into the mechanism by which ecdysone, as a precursor to the ecdysone signaling pathway, stimulates the upregulation of HIF-1α. Given that HIF-1α is predominantly induced under hypoxic conditions, we initially investigated the influence of physiological activities associated with molting on oxygen levels within the shrimp's body. Through meticulous observation and statistical analysis, we noted that the heart rate of *N*. *davidi* fluctuates across different molting stages (Fig. 4*A*). The overall molting cycle demonstrates an initial increase in heart rate followed by a subsequent decrease. Specifically, during phase C, the heart rate increased from 255 beats per minute to 331 beats per minute in phase D3; however, it subsequently declined during phases D4 and E, reaching a minimum of 222 beats per minute in phase E. Then the heart rate during phase AB exhibited a modest increase compared to phase E, approaching values observed in phase C. In contrast, gill ventilation rates displayed a decreasing trend throughout nearly all phases within stage D, with the lowest rate recorded in phase D3 (Fig. 4*B*). This observation suggested that during the early stages of molting, there was an escalation in heart rate accompanied by a deceleration in gill movement. To further elucidate the dynamics of oxygen during the molting cycle, we quantitatively mapped dissolved oxygen levels in the muscle tissue of *N*. *davidi* using fiber-optic microsensors. As illustrated in Figure 4*C*, intramuscular oxygen concentration remained at a relatively low level, demonstrating a significant decrease in comparison to the intermolt period. This low-oxygen phase exhibited temporal coordination with the pre-ecdysis transcriptional surge of hypoxia-inducible factor 1α (HIF-1α), revealing a significant correlation between endogenous oxygen concentration and molting initiation.

### The removal of gill leaves can induce extensive expression of NdHIF-1 **α**

**Figure 5:**
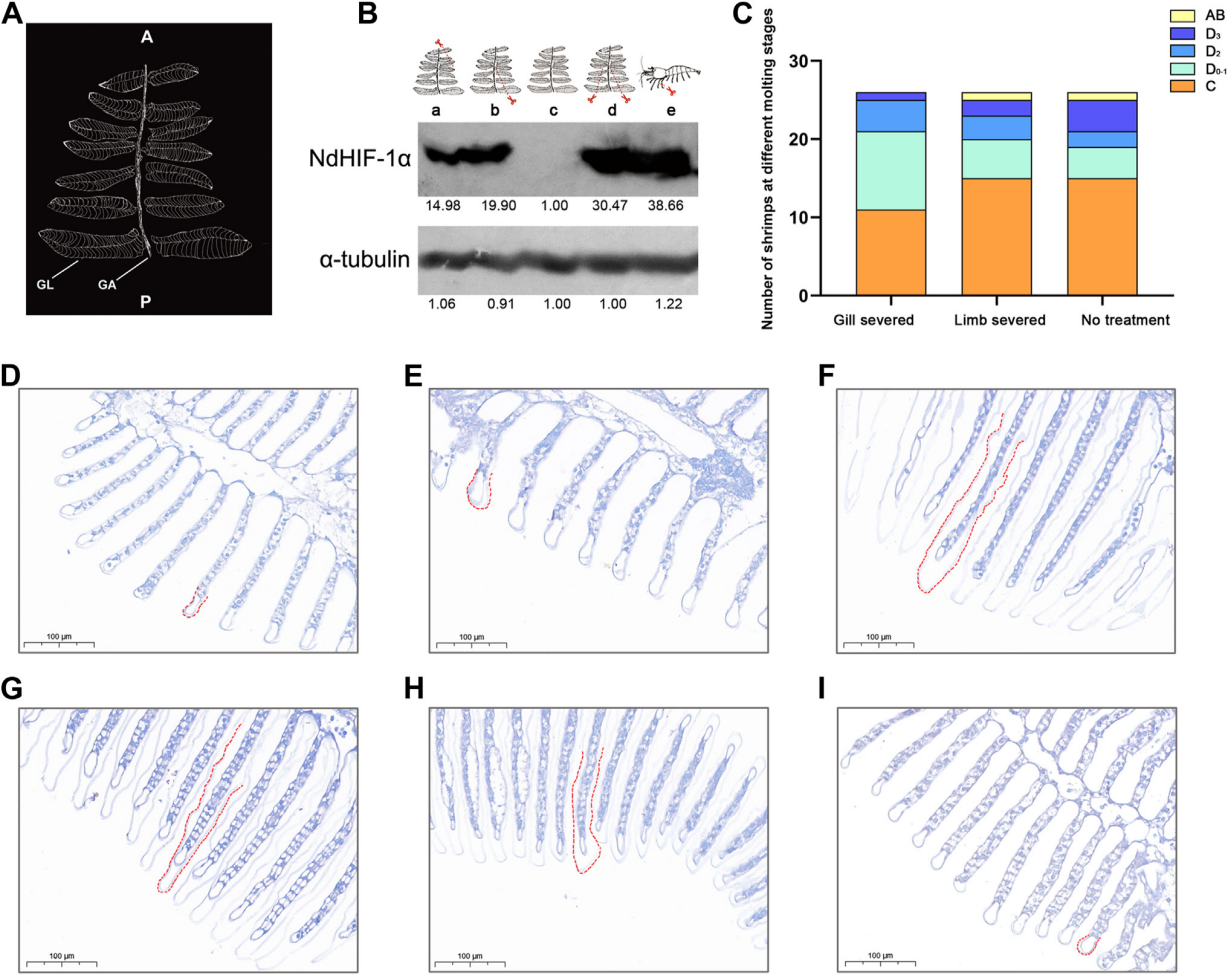
**The effect of gill leaf removal on shrimp was investigated**. *A*, the schematic of the phyllobranchiate type gill of *Neocaridina davidi*. A, anterior; P, posterior; GA, gill axis; GL, gill lamella. *B*, the Protein expression of HIF-1α in muscle under different treatments. a: unilateral anterior three gill lobes; b: unilateral posterior three gill lobes; c: control group without any treatment; d: bilateral posterior three gill lobes; e: removing the unilateral step foot. Alpha-tubulin was the internal reference protein. The numbers below blots represented the *gray* value as the fold-change relative to the control group. *C*, a statistical analysis was conducted on the molting cycles of shrimp following the removal of gills and feet. Slices of shrimp gills were prepared and stained with *toluidineblue* from various molting cycles, including the molting interval C phase (*D*), the pre-molting D0-1 phase (*E*), the pre-molting D2 phase (*F*), the pre-molting D3 phase (*G*), the pre-molting D4 phase (*H*), and the post-molting AB phase (*I*), to assess changes in the gap between the outer shell and inner epidermis.

The primary respiratory organs of *N*. *davidi* consist of seven pairs of leaf gills, which are comprised of gill axes and bilaterally attached gill leaves, as illustrated in Figure 5*A*. To simulate the potential hypoxic conditions experienced by shrimp during the pre-molting phase, certain gills were partially excised from shrimp in the inter-molting stage. Following this excision, immunoblotting was performed to assess changes in HIF expression (Fig. 5*B*). The results indicated that the experimental group—subjected to unilateral removal of the first three gill leaves, unilateral removal of the last three gill leaves, and bilateral removal of the last three gill leaves—exhibited a sequential increase in HIF-1α protein expression compared to the control group. The results showed that limb amputation at the base also enhanced HIF-1α protein expression. Subsequently, the enumeration of shrimp across various molting cycles was conducted (Fig. 5*C*). A total of 40 shrimps in each group, all in stage C, were chosen for the experimental analysis. After 3 days, deceased individuals were removed from each group, resulting in 26 shrimp remaining for observation and statistical analysis. The findings revealed that within the control group, which did not experience any mechanical injury, there were 15 shrimp present in phase C. Similarly, among those subjected to one limb excision, there were also 15 individuals observed in phase C. In contrast, only 11 individuals from the experimental group with partial gill removal were found to be in phase C. Besides, a total of 10 animals, excluding the appendage group, were currently at an early stage of molting. Within the gill removal cohort, most (15) had progressed into pre-molting stages D0-1, D3-D4 inclusive. Drawing upon previous findings suggested that the removal of gills induced inadequate oxygen supply, thereby emulating the microenvironment within the body of pre-molting shrimp. This condition subsequently triggered a significant upregulation of hypoxia-inducible factor (HIF) expression, which facilitated the molting process.

During the normal molting cycle of shrimp, did the change in gill ventilation rate lead to a slight alteration in the oxygen content within the shrimp's body, subsequently influencing HIF expression levels? We sliced and stained the gills with toluidine blue at various stages of molting. The results indicated that during stages C and D_0-1_ (Fig. 5, *D* and *E*), the outer shell layer covering the ends of the gill filaments was almost tightly adhered to the inner epidermis. As the molting cycle progressed from stages D_2_ to D_4_ (Fig. 5, *F*–*H*), this outer shell layer gradually detached from the inner epidermis, enveloping each gill filament like a coat. This detachment increased the distance for oxygen diffusion from water to the gills, thereby reducing gas exchange as external water flows through these filaments. Subsequently, as the aged shell was shed, the interstice between the gill filaments and their external environment narrowed significantly, initiating a new cycle (Fig. 5*I*). The periodic structural changes of the epidermis that envelop the gill filaments during molting might elucidate the cyclical upregulation of hypoxia-related HIF expression.

### Injection of tumor cell-free extracts promotes the molting process

**Figure 6:**
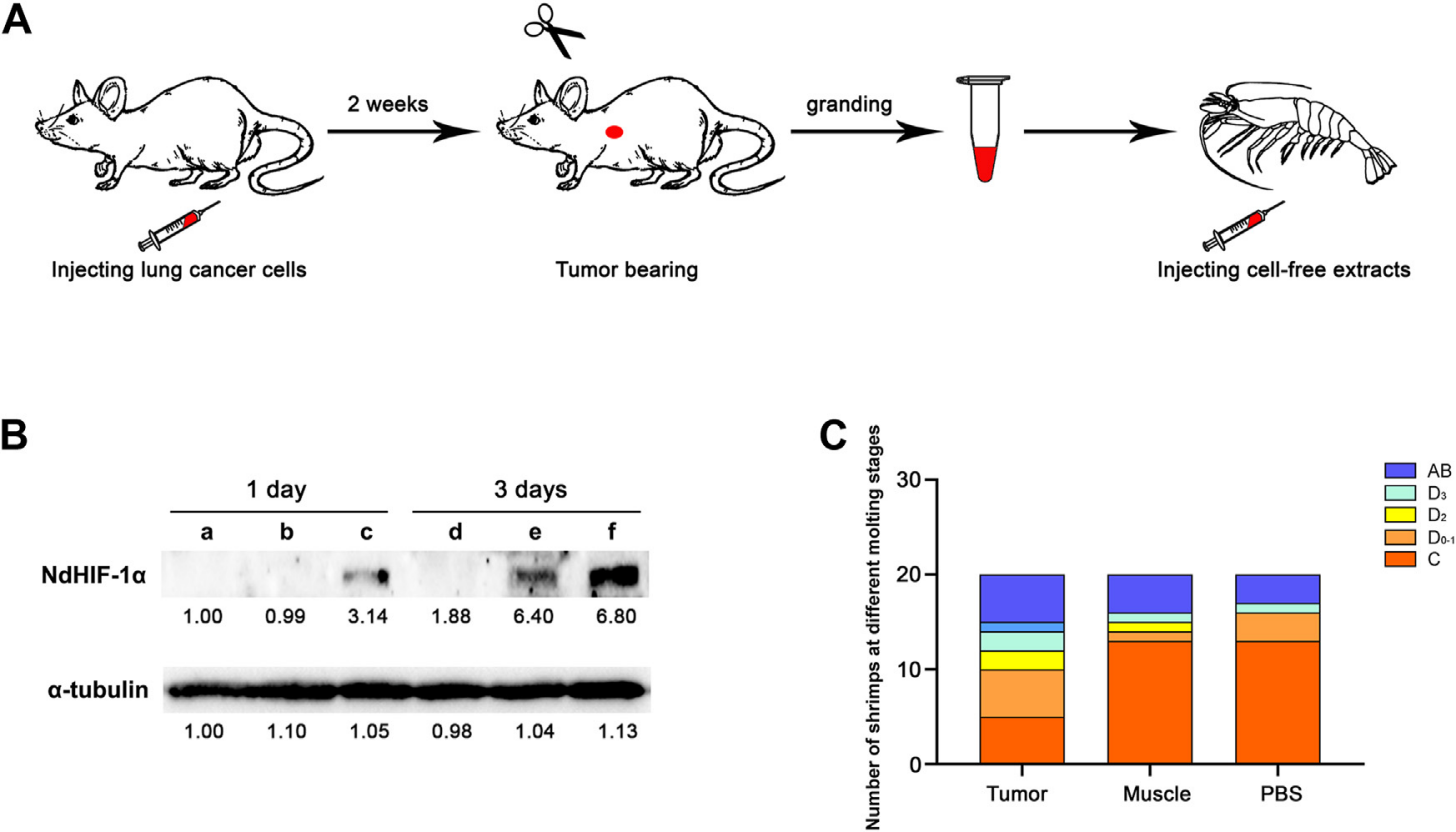
**Investigation into the impact of intramuscular injection of mouse tumor cell-free extract on shrimp HIF-1 α expression and molting dynamics**. *A*, diagrammatic representation of the experimental procedure, illustrating tumor induction and the injection of cell-free extracts. *B*, HIF-1 α protein expression in shrimp muscle was quantified at one and 3 days post-injection across PBS (a, d), mouse muscle extract (b, e), and tumor extract (c, f) treatment groups. *C*, Analysis of molting frequency 1 week following injection across the three treatment groups.

To further investigate whether alterations in the microenvironment during molting enhance the expression of HIF-1α, we employed a classic biological model of the Warburg effect—tumors. Tumor-derived acellular extracts exhibit a unique composition as a “cocktail” of tumor microenvironment-specific metabolites (*e*.*g*., lactate, ketones, oncometabolites) in physiologically optimized ratios, potentially recapitulating paracrine or other intercellular communication mechanisms observed *in vivo*. Consequently, tumor cell-free extracts were prepared and subsequently injected into shrimp to evaluate changes in HIF-1α expression. Figure 6*A* illustrated the entire experimental procedure, beginning with the extraction of tumors/muscle from mice that had developed tumors, followed by grinding and extracting these tissues before injecting them into shrimp muscle. The results indicated that HIF-1α expression levels in the shrimp significantly increased 1 day post-injection. In contrast, no notable HIF-1α expression was observed in control groups receiving PBS or muscle extracts. Three days after injection, no significant HIF-1α expression was detected in the PBS control group; however, HIF-1α expression was evident in the group that received muscle extract injections, while a substantial increase in HIF-1α expression was noted in the group administered tumor extract injections (Fig. 6*B*). Aerobic glycolysis in tumors characteristically produces elevated lactate as a hallmark metabolic byproduct. To elucidate whether lactate alone contributes to HIF-1α activation in shrimp muscle, we conducted comparative analyses of HIF-1α expression profiles in shrimp muscle under physiologically normal (simulating somatic homeostasis) and pathologically elevated (tumor-mimetic) lactate injection. Notably, neither physiological nor pathological lactate concentrations exerted significant effects on HIF-1α upregulation (Fig. S1). Collectively, these data provided evidence that the synergistic interactions among multiple metabolic intermediates and/or microenvironmental factors could lead to the upregulation of HIF-1α in shrimp muscle.

Given that preliminary results indicated a close relationship between hypoxia-inducible factor (HIF) and muscle growth, as well as various physiological metabolic processes during the pre-molting stage, we investigated whether the injection of tumor extract influenced shrimp molting. After 1 week of injections, the molting cycles of three groups of shrimps were recorded (Fig. 6*C*). In the control group receiving PBS and normal mouse muscle injections, 13 shrimp remained in phase C. In contrast, only five shrimp from the experimental group injected with tumor cell-free extract were observed to be in phase C. In the PBS injection group, four shrimp were noted to be in the pre-molting stage, while three completed molting and transitioned into the post-molting stage. In the muscle extract injection group, three shrimp were at an early stage of molting, and four had successfully completed their molt process, moving into late-stage molting. Conversely, within the experimental group injected with tumor extract, a majority (ten individuals) entered pre-molting stages: five individuals at D_0-1_; two individuals at D_2_; two individuals at D_3_; and one individual at D_4_. Additionally, five individuals completed their molt and progressed into post-molting stages.

These findings suggest that the microenvironment surrounding tumor cells can induce HIF production in shrimp, thereby regulating physiological processes to accelerate molting. Whether under natural conditions or following artificial injection of tumor extracts, HIF appears to function as a critical regulator promoting molting by modulating cellular glycolysis reactions.

## Discussion

In both vertebrates and invertebrates, glucose metabolism is intricately linked to growth, with glycolysis serving as a crucial biological process for the proliferation and development of organisms. Previous studies on the molting mechanism of *N*. *davidi* revealed that phosphofructokinase (PFK) and glycolysis played an exceptionally important role in molting-related growth (16). In this study, we analyzed PFK, HK, and PGK enzyme activities across different molt cycles in shrimp. Generally, changes in expression levels for these three enzymes followed similar patterns; all three enzymes showed significant upregulation at the onset of molting, with early D-stage expressions surpassing those observed during intermolt stages as well as both molt and late-molt stages. The activity levels for these enzymes also demonstrated a comparable trend: overall enzyme activity during premolt periods was markedly higher than during other phases. Given that PFK, HK, and PGK are key enzymes involved in glycolysis, these results indicate active muscle glycolysis throughout premolt periods. This observation aligned with research conducted on certain crabs from the genus Sphygmoides where both PFK and HK exhibited high activity levels within muscles during premolting periods, suggesting a heightened rate of glycolysis within crustacean muscles leading up to molting (30).

HIF-1 and its signaling pathway play a critical role in the growth and metabolism of animal cells. In this study, we investigated the expression of the active subunit HIF-1α in the muscles of shrimp during various molting periods. Our findings revealed that mRNA expression levels of HIF-1α during the premolt period were significantly higher than those observed in other stages, reflecting similar trends in gene expression for three key glycolytic enzymes mentioned previously. Our data revealed a dynamic pattern of HIF-1α protein expression: while peak accumulation occurs at D_4_ (Fig. 1*I*), HIF-1α levels were already significantly elevated during the D_0-1_ and D_2_ phases compared to the control group. This early-phase elevation suggests that submaximal concentrations of HIF-1α may be sufficient to activate target genes. Importantly, the transcriptional activity of HIF-1α is not strictly proportional to its protein abundance; rather, it depends on cooperative interactions with transcriptional regulators at hypoxia-response elements (HREs). Previous studies demonstrated that submaximal levels of HIF-1α can drive gene expression dependent on HREs *via* cooperative DNA binding and coactivator recruitment (31). Based on these findings, we propose that even before reaching maximal expression, early-phase HIF-1α initiates glycolytic enzyme expression pre-molt, while its D4 peak may reflect additional roles in coordinating parallel pathways (*e*.*g*., redox homeostasis (32)). Future studies aimed at dissecting the temporal coordination of the multifunctional roles of HIF-1α will further elucidate this regulatory hierarchy.

The reasons for the upregulation of HIF-1α expression during premolt were analyzed from the perspective of changes in oxygen pressure. The growth of new epidermal layers gradually detached from the inner epidermis, thereby increased the distance for oxygen diffusion from water to the gills and then reducing gas exchange. Previous studies have reported a significant increase in oxygen consumption in *Panulirus homarus* during early molting compared to inter-molting periods; however, no substantial difference was observed when comparing it with late molting stages (33). In American lobsters, oxygen consumption continues to rise throughout the premolt phase and peaks just prior to molting but experiences a sharp decline post-molting (34). In crustaceans, new epidermis formation occurs at early stages of molting while portions of the old exoskeleton are degraded and reabsorbed. Consequently, this results in a relaxed exoskeleton, where gaps form between newly developed epidermis and existing exoskeletons. And this contributes to increased diffusion distances for gill-absorbed oxygen reaching muscle cells, potentially leading to localized hypoxic conditions within muscle tissue (35). Similarly, the constitutive expression of HIF-1α is induced by intermittent tissue hypoxia as cancer cells approach their thresholds for oxygen diffusion mediated by blood vessels. (36). Hypoxia-dependent genes may also be activated under these circumstances when O_2_ supply and demand change slightly or when tissues adapt to chronic and repeated hypoxia (37). The decrease in intracellular oxygen partial pressure, which occurs due to increased oxygen consumption and reduced oxygen intake during the early stages of molting, may act as a physiological factor that contributes to the upregulation of HIF-1α expression. The specific causes and regulatory mechanisms underlying HIF-1α expression require further investigation.

The excision of gill lobes from various regions of the shrimp resulted in damage to the respiratory system, leading to a decrease in oxygen uptake capacity and an increase in HIF-1α expression. The influence of different gill lobe regions on oxygen uptake efficiency in shrimp exhibited significant variation. The differential effects of gill ablation *versus* limb amputation on molting timing (Fig. 5*C*), despite comparable upregulation of HIF-1α protein levels (Fig. 5*B*), likely stemed from distinct upstream triggers and functional prioritization. Gill removal induced systemic hypoxia by eliminating the primary respiratory organ, thereby stabilizing HIF-1α through oxygen-sensitive PHD-VHL pathways (38). In contrast, limb amputation elevated HIF-1α primarily through injury-induced inflammatory signals (*e*.*g*., ROS/NF-κB) (39), redirecting HIF-1α activity toward localized wound-healing programs rather than systemic regulation of molting. Furthermore, post-amputation osmotic stress might disrupt the crosstalk between HIF-1α and mTOR (40), uncoupling its expression from the transcriptional activation of genes related to molting. Thus, while both interventions result in elevated levels of HIF-1α, their tissue-specific induction mechanisms and downstream pathway engagements dictate divergent physiological outcomes.

Crustacean G protein-coupled receptors (GPCRs) are membrane receptors that interact with various ligands. The Y organ of the black-backed land crab secretes 5-hydroxytryptamine and octopamine, which can stimulate the synthesis of ecdysone upon binding to their GPCR receptors (41). Several GPCRs identified in the Y organ of blue crabs are differentially expressed during various molt cycles, with GPCR-B3α and GPCR-B3β being induced by ecdysone to participate in the regulation of the 20-hydroxyecdysone (20-HE) signaling pathway (42). We propose that G protein-coupled receptors (GPCRs) play a crucial role in the regulation of molting in shrimp. Pertussis toxin (PTX) can inhibit GPCR signaling, thereby limiting their involvement in molting-related pathways and impacting the efficacy of 20-hydroxyecdysone (20-HE).

The objective of this experiment was to explore the relationship between HIF-1α and the glycolytic pathway in shrimp muscle, as well as to examine the significance of this relationship in molting and rapid muscle growth. The findings indicate that elevated expression levels of HIF-1α and the upregulation of glycolysis during pre-ecdysis are essential for facilitating rapid muscle growth. We propose two primary hypotheses regarding the upregulation of glycolysis during pre-ecdysis: One may be an adaptive response to fluctuations in oxygen partial pressure or local hypoxia, while the other may serve to maintain adequate levels of glycolytic intermediates required for biosynthesis. Further investigation is warranted into the mechanisms by which HIF-1α regulates the expression of glycolytic genes throughout shrimp molting.

## Experimental procedures

### Animals

Four batches (purchased Jan/20, May/10, October/6, 2023, and March/2, 2025) of healthy shrimp were procured from a shrimp farm located in Shandong province. The shrimp were identified as *N*. *davidi* through a combination of morphological analyses (including body shape, coloration, and appendage structure) and mitochondrial COI gene sequencing. The sequences exhibited ≥99% identity to the reference entry (GenBank: LC830199.1). Subsequently, shrimp from all batches were co-reared within the shrimp recycling system present in the laboratory. To facilitate aeration and eliminate chlorine, tap water was introduced into the glass cylinder, followed by a continuous inflation process utilizing air pumps, which lasted for 24 h. The concentration of dissolved oxygen in the water was monitored using a dissolved oxygen meter (Seven2Go pro S9 DO, METTLER TOLEDO) to ensure that it remained within the range of 6 to 8 mg/l. All subsequent experimental procedures will be conducted to ensure that the dissolved oxygen levels in both the control and experimental group tanks remain consistent. It is essential to maintain the water temperature at approximately 24 °C. For illumination purposes, full-spectrum LED lights were employed, adhering to a lighting cycle of 4 h of illumination followed by 20 h of darkness. The shrimp were fed with a commercial diet (Shrimp King Complete, Dennerle) consisting of insect proteins, dandelion, stinging nettles, spinach, mulberry leaves, chlorella, montmorillonite, moringa oleifera, rosemary, mannan oligosaccharides, β-glucans, flower pollen, turmeric, and cinnamon. All groups were provided with an identical quantity of feed, amounting to 5% of their body weight per day. Feeding occurred twice daily, in the morning and evening. After an acclimation period of 1 week for newly introduced shrimp, an experiment was conducted. Shrimp exhibiting consistent body length and vitality were selected and observed under a stereomicroscope. Molting stages were determined based on the microstructural changes observed in the seta wall, seta cavity, and epidermal cells of the tail fan of *N*. *davidi*. During dissection on ice, 4 to 6 shrimp muscles were collected as samples, frozen in liquid nitrogen, and subsequently stored in a refrigerator at −80 °C.

### Real-time quantitative (RT-qPCR)

QPCR was conducted on real-time PCR detection system (LightCycler480, Roche) using AceQ qPCR SYBR Green Master Mix (Cat#Q111, Vazyme). Ndβ-actin was served as an internal reference gene and amplified in parallel with target genes to calculate the relative expression levels of the target genes. The cDNA sequences of HIF-1α, PFK, PGK, and HK have been deposited in GenBank under the accession numbers OR996017, OR996020, PV590104, and OR996019, respectively. The primers for the target genes are presented in Table S1. The entire muscle mass of 3 shrimp in each molting stage or time point was utilized for RNA extraction and cDNA synthesis, serving as the template in triplicate qPCR reactions.

### Detection of glycolysis extent in muscles during different molting stages

The muscle tissues of shrimp at each stage were ground using a homogenizer, and the enzyme activities of NdPFK, NdHK, and NdPGK in the muscles of seven molting stages were detected according to the instructions of PFK, HK, and PGK enzyme-linked immunosorbent assay kit (Cat#A091489, A091485, A091450, Shanghai Fusheng Industrial Co., Ltd). According to the instructions of the testing kit (Cat#BC2505, BC2235, Solarbio), the glucose and lactate content were measured using a trace method.

### Western blot analysis

The total protein samples were loaded for each lane on sodium dodecyl sulfate polyacrylamide gel electrophoresis (SDS-PAGE) and subsequently transferred onto a polyvinylidene difluoride membrane (Cat#IPVH00010, Millipore). The membrane was then probed with indicated primary antibodies against HIF-1α (1:1000, Cat#ABIN2779347, Antibodies-online GmbH, predicted protein size: 115kD), α-tubulin (1:5000, Cat#AT819, Beyotime, predicted protein size: 55kD) HK1 (1:1000, Cat#A23524, ABclonal, predicted protein size: 90kD), PFK (1:2000, Cat#A5477, ABclonal, predicted protein size: 108kD), and PGK1 (1:5000, Cat#68035-1-Ig, proteintech, predicted protein size: 45kD). Signals were detected using HRP Chemiluminescent Substrate (Cat#34577, Thermo Scientific), and the exposed film images were analyzed with ImageJ and Photoshop software.

### Knockdown of NdHIF-1**α** by RNA interference and efficiency detection

Based on the open reading frame sequence of NdHIF-1α gene, specific primers (Table S1) containing EcoR I and Xba I restriction cleavage sites were designed for the synthesis of double-stranded RNA (dsRNA). This was achieved through vector expression in *Escherichia coli*, and the quality of the dsRNA was verified using agarose gel electrophoresis. In the injection experiment, shrimp at the intermolt stage, approximately 1.2 cm in body length, were randomly divided into two groups: an experimental group and a control group. Each shrimp received a microinjection of either 400 nl dsRNA or an equivalent volume of ultra-pure water at the junction of cephalothoracic armor using a micro-injection system (WPI, Sarasota, FL, USA). At three time points—1 day, 3 days, and 7 days post-injection—muscle samples from three shrimp were pooled for total RNA extraction to perform quantitative PCR and total protein extraction for Western blot analysis to assess interference efficiency.

### Relative expression analysis of NdPFK, NdHK, and NdPGK in the context of NdHIF-1**α** knockdown and statistics on the number of molting cycles completed

The transcription levels of three glycolytic enzymes—NdPFK, NdHK, and NdPGK—in the muscle tissues of shrimp subjected to silencing of NdHIF-1α were examined alongside a control group at 1, 3, and 7 days post-injection. Additionally, 18 shrimp from each group were housed in mesh isolation boxes within the same glass tank under identical feeding conditions. The shed exoskeletons were collected and counted daily; subsequently, the number of molting cycles completed over 1 month was calculated.

### Injection of 20-hydroxyecdysone (20-HE) and GPCR inhibitor

The shrimp at the intermolt stage, approximately 1.2 cm in body length, were randomly divided into three groups, with 12 shrimp in each group. Muscle tissue at the junction of the cephalothoracic armor of each shrimp was injected with either 400 nl of a solution containing 200 μg/ml of 20-hydroxyecdysone (20-HE; Cat#505654, Sangon Biotech), or a mixture comprising 1 ng of GPCR inhibitor pertussis toxin (PTX; Cat#181, List Labs) and 200 μg/ml of the same reagent. Control groups received ultrapure water *via* a micro-injection system (WPI). The three groups of shrimps were housed in mesh isolation boxes within the same glass tank under identical feeding conditions. Eight hours post-injection, while the shrimp remained in the intermolt stage, muscle samples were pooled from four individuals per group and subsequently stored at −80 °C for total RNA extraction to be used in quantitative PCR analysis.

### Removing the gill lobes of *N*. *davidi*

Shrimp at the intermolt stage, approximately 2.5 cm in body length, were selected and randomly divided into five groups, each containing 12 shrimp. Prior to the experimental procedures, the shrimp were placed on ice for about 2 min to minimize physical stimulation and prevent excessive movement, thereby facilitating the operation. The following treatments were conducted under a microscope: removal of three anterior gill lobes unilaterally, removal of three posterior gill lobes unilaterally, and removal of three posterior gill lobes bilaterally. One control group underwent unilateral step foot removal, while another group received no treatment. 12 hours post-treatment, muscle samples from three shrimp were pooled together for total protein extraction for Western blot analysis.

### Heart rate, gill ventilation rate, and dissolved muscle oxygen concentration of *N*. *davidi* among different molting stages

Healthy and vigorous shrimp at various molting stages (n = 6), approximately 1.2 cm in body length, were selected for the study. Videos of heartbeats and gill movements were recorded under a microscope and subsequently analyzed using an electronic counter to calculate the rates of heart activity and gill ventilation. The oxygen content within the muscle tissue of shrimp at different molting stages was measured using a PreSens OXY-1 ST TRACE (Germany), with data logged at 5-s intervals. A needle-type oxygen microsensor (PM-PSt7) was inserted 3 mm deep into the abdominal muscle tissue through the arthrodial membrane at the cephalothorax-abdomen junction of the shrimp, avoiding calcified exoskeletal regions. After allowing sufficient time for data stabilization, readings were recorded. For most molting stages (C, D_0-1_, D_2_, D_3_, D_4_ and AB), 15 shrimps were established for oxygen measurements. However, due to the transient nature of ecdysis (E stage), only 9 individuals were successfully captured.

### Preparation and injection of tumor cell-free extract

LLC (Lewis lung carcinoma) cells are commonly utilized as a model for studying the mechanisms of cancer development. Due to their high tumorigenicity, we selected LLC cells as the tumor cell line for injection into mice. A suspension of 5 × 10^∧^7 LLC cells was subcutaneously inoculated into C57BL/6 mice aged 4 to 6 weeks. After 2 weeks, the mice were euthanized to extract tumor tissue and left hind leg muscle tissue. The obtained tissues were rapidly frozen in liquid nitrogen for 2 min. Subsequently, the tissues were homogenized in phosphate-buffered saline (PBS) using a disposable grinding pestle, resulting in a coarse homogenate with a concentration of 33% weight/volume. To begin, the crude homogenate was subjected to centrifugation at a force of 500×*g* for a duration of 10 min, during which any substantial debris was discarded and the resulting supernatant collected. Following this step, the aforementioned supernatant was subjected to centrifugation at a rate of 10,000×*g* for a period of 10 to 15 min, after which the supernatant was again collected. Subsequently, the collected supernatant was exposed to irradiation under a UV lamp for a duration of 20 s, thereby yielding the cell-free extract from lung cancer subcutaneous tumors in mice.

Healthy and energetic shrimp, measuring approximately 1.2 cm in body length, were selected for the study. The tail tips of these shrimp were examined under a posture microscope to determine their molting period. Subsequently, the shrimp were randomly assigned to three distinct groups: one group for mouse lung cancer subcutaneous tumor treatment, another group for left hind leg muscle tissue analysis, and a control group treated with PBS. Each group comprised 60 shrimp. The shrimp were placed in culture dishes, and the aforementioned supernatants were injected into the muscles at the junction of their head and sternum using a microinjection system, with an injection volume of 800 nl per shrimp. Following the completion of all injections, the shrimp were divided into three groups and housed in isolation boxes measuring 25 × 15 × 15 cm within a shared tank environment. They received an equal quantity of commercial shrimp food daily. On Days 1, 3, and 5 post-injection, muscle tissue samples from the shrimp were extracted for Western blot analysis while simultaneously monitoring their remaining molting cycle.

## Data availability

All data generated or analyzed during this study are included in this article and its supplementary information files.

## Supporting information

This article contains supporting information.

## Conflict of interest

The authors declare that they have no conflicts of interest with the contents of this article.
