# Supplementary material for: Hypoxia-inducible factor-1 alpha modulates muscle growth and the molting process through its regulation of glycolysis in Neocaridina davidi
Source: J Biol Chem. 2025 May 27;301(7):110298. doi: 10.1016/j.jbc.2025.110298 (PMC12221361; doi:10.1016/j.jbc.2025.110298)
Supplement: Figure S1 [file mmc1.docx]

**
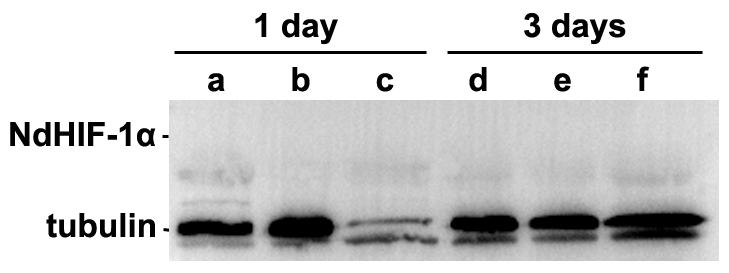
**

**Supplemental Fig. 1.** The control group received an injection of PBS (a, d), while the experimental groups were administered 0.047 μg (b, e) and 0.95 μg (c, f) of lactic acid, respectively, with each shrimp receiving an injection volume of 800nl. Muscle samples from three shrimp were collected at one day and three days post-injection for Western blot analysis.
