# Supplementary material for: Hypoxia-inducible factor-1 alpha modulates muscle growth and the molting process through its regulation of glycolysis in Neocaridina davidi
Source: J Biol Chem. 2025 May 27;301(7):110298. doi: 10.1016/j.jbc.2025.110298 (PMC12221361; doi:10.1016/j.jbc.2025.110298)
Supplement: Table S1 [file mmc2.docx]

**Table S1 Primers used in this study**

The underlined regions represent the recognition sequences of restriction endonucleases. F, forward; R, reverse.

| Primer name | Primer sequence (5'-3') | Used for |
| --- | --- | --- |
| Ndβ-actin F | TGTGACGATGAAGTAGCAGCA | real-time PCR |
| Ndβ-actin R | AATCTTTCTGACCCATTCCAAC | real-time PCR |
| HIF-1α-qF | TGAGGTGATGGCAGATGGAACT | real-time PCR |
| HIF-1α-qR | CCAGGCGATGACTGAAATAAGG | real-time PCR |
| NdPFK-qF | ACTGCTTATTCTCATCAACGCTGCTT | real-time PCR |
| NdPFK-qR | ACCTCTCCGATTCCAACTTCTCACA | real-time PCR |
| NdHK-qF | ACTCAACGATACCACAGGCACA | real-time PCR |
| NdHK-qR  NdPGK-qF  NdPGK-qR | TCATCCATAGCACCATCCCAC  AACAACATCGGGACGGAACG  TGATGCGAGCAGAGCAGGAG | real-time PCR  real-time PCR  real-time PCR |
| dsHIF-1α-F | GCTCTAGAAAGAAGTGCGAAGGGAGTGA | RNAi |
| dsHIF-1α-R | GGAATTCAGTGGAAGTGGGCAAGGATT | RNAi |
